# Investigation of changes in ankylosing spondylitis disease activity through 2021 COVID-19 wave in Taiwan by using electronic medical record management system

**DOI:** 10.1038/s41598-023-27657-6

**Published:** 2023-01-07

**Authors:** Pei-Ju Huang, Yun-Wen Chen, Tsai-Hung Yen, Yen-Tze Liu, Shih-Ping Lin, Hsin-Hua Chen

**Affiliations:** 1 Department of Family Medicine, Changhua Christian Hospital, Changhua, Taiwan; 2 Division of General Medicine, Department of Internal Medicine, Taichung Veterans General Hospital, 1650 Taiwan Boulevard Sect. 4, Taichung, 40705 Taiwan, ROC; 3 Division of Allergy, Immunology and Rheumatology, Department of Internal Medicine, Taichung Veterans General Hospital, Taichung, Taiwan; 4 Department of Post-Baccalaureate Medicine, College of Medicine, National Chung Hsing University, Taichung, Taiwan; 5 Division of Infection, Department of Internal Medicine, Taichung Veterans General Hospital, Taichung, Taiwan; 6 School of Medicine, National Yang-Ming Chiao Tung University, Taipei, Taiwan; 7 Department of Industrial Engineering and Enterprise Information, Tunghai University, Taichung, Taiwan; 8 Institute of Biomedical Science and Rong Hsing Research Center for Translational Medicine & Big Data Center, Chung Hsing University, Taichung, Taiwan

**Keywords:** Autoimmunity, Infectious diseases, Diseases, Medical research, Rheumatology

## Abstract

We aim to investigate the alteration in disease activity of ankylosing spondylitis (AS) individuals before, during, and after the COVID-19 wave in Taiwan by using electronic medical-record management system (EMRMS). We identified 126 AS individuals from the single center, and gathered data of the three disease activities (Bath Ankylosing Spondylitis Disease Activity Index [BASDAI], *Ankylosing Spondylitis Disease Activity Score with erythrocyte sedimentation rate [*ASDAS-ESR], and *Ankylosing Spondylitis Disease Activity Score with* C-Reactive Protein [ASDAS-CRP]) by using EMRMS before (7 February to 1 May, 2021), during (2 May to 24 July, 2021), and after the COVID-19 wave (25 July to 16 October, 2021). We compared the disease activity measures of the three phases through a paired *t* test. Among the 126 individuals, CRP was significantly higher during the COVID-19 wave (0.2 (0.1, 0.5) mg/dl, p = 0.001) than before the wave (0.2 (0.1, 0.4) mg/dl), ESR (8.0 (4.0, 15.0) mm/h, p = 0.003) and ASDAS-ESR (1.4 (1.0, 1.9), p = 0.032) were significantly higher after the wave than during the wave (6.0 (3.0, 12.0) mm/h and 1.2 (0.9, 1.8) mm/h) e. ESR, CRP, ASDAS-ESR and ASDAS-CRP were all significant higher after COVID-19 wave than before. The disease activities of AS individuals in Taiwan worsened after 2021 COVID-19 wave in Taiwan.

## Introduction

Ankylosing spondylitis (AS) is a common rheumatological disease. It is a form of axial spondyloarthritis (axSpA) that is characterized by chronic back pain with articular and periarticular extraspinal symptoms. AS is indicated by the human leukocyte antigen (HLA)-B27, which is either positive or negative. In AS, the C-reactive protein (CRP) or erythrocyte sedimentation rate (ESR) is elevated, and typical sacroiliitis and spinal abnormalities are indicated by radiographs^1^. AS treatment strategies, including regular monitoring of disease activity, are generally applied by rheumatologists according to the 2016 recommendations of the Assessment of SpondyloArthritis International Society and European League Against Rheumatism^2^. The Ankylosing Spondylitis Disease Activity Score (ASDAS) and Bath Ankylosing Spondylitis Activity Disease Activity Index (BASDAI) are both implemented to evaluate disease activity in individuals with AS through the use of a self-administered questionnaire, with or without clinical laboratory data. Both ASDAS and BASDAI were reported to perform similarly well when used to differentiate between high and low disease activity in individuals with axPsA^3^.

COVID-19 originated in Wuhan in January 2019 and is caused by the severe acute respiratory coronavirus 2 (SARS-CoV-2). As of the time of writing, the COVID-19 pandemic is still ongoing. In 2020, the impact of the pandemic in Taiwan was lower than in most other industrialized countries, with a total of seven deaths^4,5^. However, a sharp surge in cases occurred in early May 2021 after an outbreak among Taiwanese crew members of the state-owned China Airlines in late April 2021^6^. On July 23, 2021, the Taiwan Centers for Disease Control eased COVID-19 restrictions considering the gradual stabilization of the domestic COVID-19 situation^7^.

Recent studies have reported that avoidance of doctor visits, laboratory testing, and the use of telehealthcare have been more common since the COVID-19 pandemic^8,9^. COVID-19 was reported to negatively affect the disease activity and psychological state of individuals with AS in Turkey, US, and New Zealand^10–12^. A Swiss study reported that patients had a stable and slightly decreased BASDAI during the COVID-19 wave and slightly increased BASDAI after the COVID-19 wave^8^.

An electronic medical-record management system (EMRMS) was implemented at the Taichung Veterans General Hospital (TCVGH) in November 2016 for patients with AS. The ASDAS and BASDAI of patients are routinely evaluated using the EMRMS; in this system, patient disease activity data are automatically integrated with ESR/CRP data. We aimed to assess the variations in the disease activity of patients with AS during the 2021 COVID-19 wave in Taiwan.

## Methods

### Ethics statement

The Institutional Review Board (I) of Taichung Veterans General Hospital approved the study (TCVGH-IRB No.: CE22171A) and waived informed consent because all data were anonymized before analysis, and all experiments were performed in accordance with relevant guidelines and regulations.

### Study design

This was a single-center retrospective cohort study.

### Data sources

The EMRMS was established in November 2016 to assist rheumatologists in conducting ASDAS and BASDAI assessments and comprehensively evaluating clinical outcomes in all patients with AS at TCVGH. The EMRMS database contains information on, for example, AS-related biological characteristics (CRP level and ESR), patient comorbidities, patient history, and family history. The reliability and validity of the data have been verified^13^. Patients with AS were consecutively enrolled in the TCVGH-AS cohort after they were diagnosed with AS by a TCVGH rheumatologist according to the modified New York criteria of 1984^14^. CRP and ESR data were automatically uploaded to the TCVGH healthcare information system to reduce human error. The information collected by trained nurses included those on clinical characteristics, onset age, comorbidities at presentation (hypertension, diabetes mellitus, hyperlipidemia, hepatitis B, hepatitis C, renal insufficiency, gout, coronary artery disease, stroke, periodontal disease, osteoporosis, and tuberculosis), periarticular extraspinal conditions (synovitis, enthesitis, and dactylitis) and nonarticular manifestations (psoriasis, uveitis, and IBD), family history of autoimmune disease, and patient history of arthropathy. These data were obtained through standardized questionnaires and worksheets to ensure reproducibility in accordance with good laboratory practice. The rheumatologist in charge then confirmed the patients’ clinical characteristics, and the nurses assisted the patients to complete the self-assessment questionnaires for disease evaluation. The questionnaires were completed on the TCVGH app during 3-month outpatient visits or on blood examination days.

### Study populations

From February 7, 2021 to October 16, 2021, 342 patients with AS were monitored using the EMRMS; 252 patients were ever assessed in pre COVID-19 phase (BASDAI, ASDAS-ESR, or ASDAS-CRP)\; 126 patients were assessed for at least one disease activity during each study period, irrespective of whether the assessment was performed during consultations or telehealthcare or using the EMRMS web application. The patients at TCVGH started using the EMRMS app in November 2016 to monitor disease activity and drug compliance every 3 months.

### Periods of disease activity assessments

**Figure 1:**
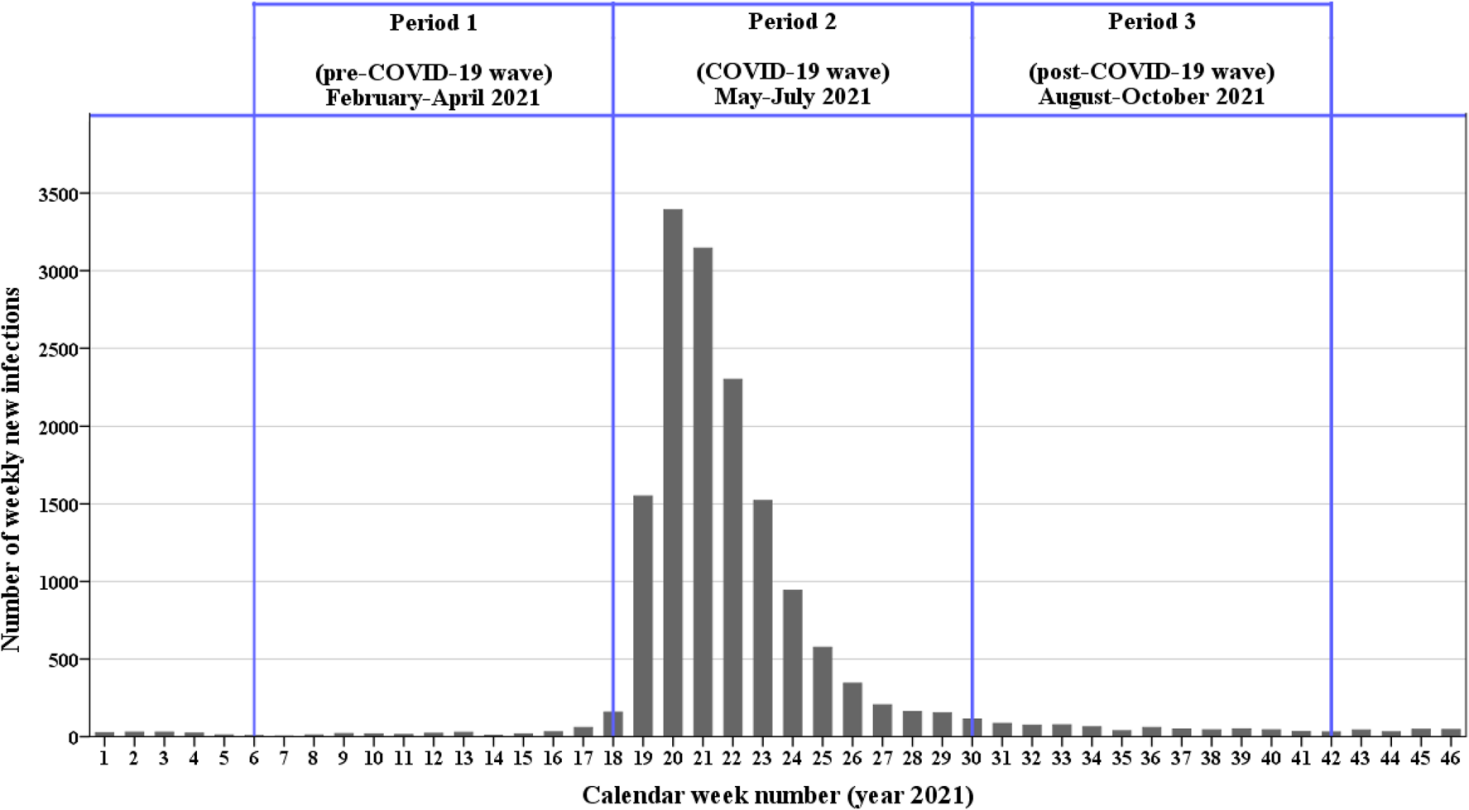
Weekly numbers of new SARS-CoV-2 infections registered in Taiwan; pre-COVID-19-wave phase: 6th–17th week of 2021 (February 7–May 1, 2021). COVID-19-wave phase: 18th–29th week of 2021 (May 2–July 24, 2021) post-COVID-19-wave phase: 30th–41st week of 2021 (July 25–October 16, 2021). *The weekly calculations are based on WHO’s year-week comparison table, where each week includes all 7 days and the first week of each year does not necessarily include January.

The weekly numbers of new SARS-CoV-2 infections in 2021 in Taiwan are depicted in Fig. 1.

The longitudinal course of SARS-CoV2-infection case numbers was divided into three phases: (1) the pre-COVID-19-wave phase (7 February to 1 May, 2021), (2) the COVID-19-wave phase (2 May to 24 July, 2021), and (3) the post-COVID-19-wave phase (25 July to 16 October, 2021).

### Disease activity assessments

Different versions of the ASDAS were combined with patient-oriented measures (global assessment of disease activity, back pain, duration of morning stiffness, and peripheral pain or swelling; all parameters were rated using a numerical rating scale [NRS] of 0–10) with a laboratory measure of inflammation (CRP or ESR level)^15^.The BASDAI was based on only patient-oriented measures (six questions, including the degree of fatigue/tiredness experienced, AS-related pain in the neck, back, or hip, pain or swelling in other joints, discomfort in any tender areas caused by touch, pressure and discomfort since waking up, and the duration of morning stiffness since waking up; all parameters were rated using a NRS of 0–10). Patient-reported outcomes (PROs) provide key assessments of function and the ability to perform daily activities. PROs aid the assessment of responses to treatment through physician-assessed clinical measures^16,17^.

### Statistical analysis

Continuous variables are reported as means ± standard deviation (SD), and categorical variables are reported as percentages. Differences in continuous variables were assessed for the same patient at two time points by using a paired *t* test. Kolmogorov–Smirnov test was used for normality test, non-parametric data was used Wilcoxon signed rank test to compare paired data. The ASDAS assessment was used to measure four disease activity states: inactive, moderate, high, and very high. Disease activity was evaluated according to three cutoff values: 1.3, 2.1 and 3.5 units for ASDAS-CRP and ASDAS-ESR, and the optimal BASDAI values were 2.1, 3.1 and 3.7 units corresponding to ASDAS-CRP; 2.0, 2.6, and 4.8 units corresponding to ASDAS-ESR in Taiwanese population^18^. The following cutoff values were selected to indicate improvement: a change of ≥ 1.1 units indicated a significant clinical improvement, and a change of ≥ 2.0 units indicated a major improvement^19^. The results of a cross-validation analysis strongly supported the selected cutoff values. The data were analyzed using SAS software (SAS Institute, Cary, NC, USA).

### Ethical standards

Ethics approval was obtained according to the policy of the Ethics Committee of the Taichung Veterans General Hospital (TCVGH-IRB No.: CE21149B).

## Results

As displayed in Table 1, 126 patients were assessed for at least one disease activity during each study period; the mean age of the patients was 43.2 years, with an SD of 11.3 years; 24.6% were female; 87.3% were HLA-B27 positive; the mean disease duration was 18.1 years, with an SD of 10.7 years; and 25.4% were treated with biologics. Table 1 also displays data for various comorbidities, such as hypertension, diabetes, hyperlipidemia, hepatitis B, hepatitis C, chronic renal failure, gout, coronary artery disease, stroke, periodontal disease, osteoporosis, and tuberculosis, and AS symptoms and for family history and patient history.

**Table 1:** Baseline characteristics obtained using the electronic medical record management system (EMRMS) before and after the COVID-19 wave in the selected patients with AS.

	AS patients used EMRMS	Ever assessed at any phase	Assessed in all three periods	p value
	n = 342	n = 252	n = 126	
**Age, years (Mean ± SD) at outbreak day**	43.8 ± 12.2	43.2 ± 11.7	43.2 ± 11.3	0.818
**Gender**				
Female	91 (26.6)	70 (27.8)	31 (24.6)	0.805
Male	251 (73.4)	182 (72.2)	95 (75.4)	
**Disease duration, years (Mean ± SD) at outbreak day**	17.4 ± 10.2	17.2 ± 10.0	18.1 ± 10.7	0.742
**Biologics (12 weeks before outbreak)**				
Etanercept	7 (2.0)	7 (2.8)	5 (4.0)	0.509
Adalimumab	26 (7.6)	24 (9.5)	12 (9.5)	0.656
Golimumab	24 (7.0)	24 (9.5)	15 (11.9)	0.218
Secukinumab	14 (4.1)	14 (5.6)	9 (7.1)	0.388
Ustekinumab	0 (0.0)	0 (0.0)	0 (0.0)	NA
Certolizumab	1 (0.3)	1 (0.4)	0 (0.0)	0.786
**HLA-B27**	300 (87.7)	220 (87.3)	110 (87.3)	0.986
**Co-morbidities**				
Hypertenion	57 (16.7)	39 (15.5)	19 (15.1)	0.885
Diabetes mellitus	25 (7.3)	16 (6.3)	9 (7.1)	0.897
Hyperlipidemia	46 (13.5)	34 (13.5)	18 (14.3)	0.971
Hepatitis B	31 (9.1)	20 (7.9)	10 (7.9)	0.863
Hepatitis C	8 (2.3)	4 (1.6)	2 (1.6)	0.766
Renal insufficiency	7 (2.0)	6 (2.4)	4 (3.2)	0.775
Gout	10 (2.9)	6 (2.4)	2 (1.6)	0.706
Coronary artery disease	5 (1.5)	4 (1.6)	1 (0.8)	0.814
Stroke	2 (0.6)	0 (0.0)	0 (0.0)	0.330
Periodontitis	61 (17.8)	46 (18.3)	21 (16.7)	0.929
Osteoporosis	22 (6.4)	19 (7.5)	10 (7.9)	0.803
Tuberculosis history	14 (4.1)	11 (4.4)	3 (2.4)	0.619
**AS symptoms**				
Uveitis	83 (24.3)	61 (24.2)	27 (21.4)	0.797
Psoriasis	20 (5.8)	14 (5.6)	5 (4.0)	0.723
Crohn’s disease	0 (0.0)	0 (0.0)	0 (0.0)	NA
Ulcerative colitis	3 (0.9)	2 (0.8)	2 (1.6)	0.737
Peripheral arthritis	62 (18.1)	49 (19.4)	30 (23.8)	0.388
Enthesitis	62 (18.1)	47 (18.7)	24 (19.0)	0.971
Dactylitis	6 (1.8)	5 (2.0)	3 (2.4)	0.908
**Family history**				
AS-First degree relatives	59 (17.3)	43 (17.1)	21 (16.7)	0.989
AS-Secondary degree relatives	84 (24.6)	62 (24.6)	32 (25.4)	0.981
Psoriasis	15 (4.4)	10 (4.0)	5 (4.0)	0.962
Psoriatic arthritis	3 (0.9)	3 (1.2)	2 (1.6)	0.801
Uveitis	16 (4.7)	14 (5.6)	5 (4.0)	0.777
Crohn’s disease	2 (0.6)	2 (0.8)	0 (0.0)	0.616
Ulcerative colitis	3 (0.9)	3 (1.2)	0 (0.0)	0.483
Rheumatoid arthritis	21 (6.1)	11 (4.4)	3 (2.4)	0.221
Systemic lupus erythematosus	12 (3.5)	6 (2.4)	4 (3.2)	0.730
Sicca syndrome	10 (2.9)	8 (3.2)	3 (2.4)	0.911
**Past history**				
Total hip replacement	7 (2.0)	4 (1.6)	3 (2.4)	0.855
Total knee replacement	0 (0.0)	0 (0.0)	0 (0.0)	NA
Fracture	32 (9.4)	24 (9.5)	13 (10.3)	0.951
Palindromic rheumatism	2 (0.6)	2 (0.8)	2 (1.6)	0.569

*AS* ankylosing spondylitis, *ASDAS* ankylosing spondylitis disease activity score.

### Number of assessments

After Wilcoxon Signed Rank Test, compared with that in the pre-COVID-19 wave phase, the number of assessments using EMRMS was significantly decreased in the cohort of all AS patients used EMRMS (0.0 (− 1.0, 0.0), p = 0.002; 0.0 (− 1.0, 0.0), p = 0.029) and cohort of ever assessed disease activities at any phases on EMRMS (0.0 (− 1.0, 0.0), p < 0.001; 0.0 (− 1.0, 0.0), p < 0.001) during or after COVID-19 wave compared with pre-COVID-19 wave. The number of ASDAS/BASDAI assessments in EMRMS app during three phases was not significantly change in the cohort of assess in all three phases on EMRMS (Table 2).

**Table 2:** Numbers of ASDAS/BASDAI assessments in EMRMS app.

	Pre-COVID-19-wave phase (A)	COVID-19-wave phase (B)	Post-COVID-19-wave phase (C)	(B)-(A)	p value^#^	(C)-(B)	p value^#^	(C)-(A)	P value^#^
AS patients used EMRMS, n = 342	1.0 (0.0, 1.0)	1.0 (0.0, 1.0)	1.0 (0.0, 1.0)	0.0 (− 1.0, 0.0)	0.002	0.0 (0.0, 1.0)	0.351	0.0 (− 1.0, 0.0)	0.029
Ever assessed in pre COVID-19 phase, n = 252	1.0 (1.0, 1.0)	1.0 (0.0, 1.0)	1.0 (0.0, 1.0)	0.0 (− 1.0, 0.0)	< 0.001	0.0 (0.0, 0.0)	0.780	0.0 (− 1.0, 0.0)	< 0.001
Assessed in all three phases, n = 126	1.0 (1.0, 2.0)	1.0 (1.0, 2.0)	1.0 (1.0, 2.0)	0.0 (0.0, 0.0)	0.791	0.0 (0.0, 0.0)	0.629	0.0 (0.0, 0.0)	0.858

All patients with AS were assessed using the EMRMS between Feb 7 and Oct 16, 2021.

Pre-COVID-19-wave phase: 6th–17th week of 2021 (February 7–May 1, 2021).

COVID-19-wave phase: 18th–29th week of 2021 (May 2–July 24, 2021).

Post-COVID-19-wave phase: 30th–41st week of 2021 (July 25–October 16, 2021).

*The weekly calculations are based on WHO’s year-week comparison table, where each week includes all 7 days and the first week of each year does not necessarily include January 1.

^#^Kolmogorov–Smirnov p value < 0.05, Wilcoxon Signed Rank Test is used.

### Disease activity before, during, and after the COVID-19 wave in patients with AS

ASDAS and BASDAI were combined PROs with or without a laboratory measure of inflammation. After Wilcoxon signed rank test, compared with the pre-COVID-19 phase, the COVID-19 phase had a significantly higher CRP (0.2 (0.1, 0.5) vs. 0.2 (0.1, 0.4) mg/dL, p = 0.001). Compared with the COVID-19 phase, the post-COVID-19 phase had significantly higher ESR (8.0 (4.0, 15.0) vs. 6.0 (3.0, 12.0) mm/h, p = 0.003) and ASDAS-ESR (1.4 (1.0, 1.9) vs. 1.2 (0.9, 1.8), p = 0.032), but lower BASDAI (1.5 (0.6, 2.6) vs. 1.6 (0.6, 2.8), p = 0.024). Compared with those in the pre-COVID-19 phase, ESR, CRP, ASDAS-ESR and ASDAS-CRP were significantly higher in the post-COVID-19 phase. Most of PROs symptoms are not significantly change, however, the symptom of fatigue was significantly higher in COVID-19 phase (Table 3).

**Table 3:** Disease activity scores of patients with AS assessed in all three phases (n = 126).

	Pre-COVID-19-wave phase (A)	COVID-19-wave phase (B)	Post-COVID-19-wave phase (C)	(B)-(A)	p value^#^	(C)-(B)	p value^#^	(C)-(A)	p value^#^
ESR (mm/h)	6.0 (3.0, 12.0)	6.0 (3.0, 12.0)	8.0 (4.0, 15.0)	0.0 (− 1.0, 2.0)	0.284	1.0 (− 1.0, 4.0)	0.003	1.0 (− 1.0, 5.0)	< 0.001
CRP (mg/dL)	0.2 (0.1, 0.4)	0.2 (0.1, 0.5)	0.2 (0.1, 0.5)	0.01 (− 0.02, 0.2)	0.001	0.0 (− 0.1, 0.1)	0.272	0.02 (− 0.03, 0.1)	< 0.001
ASDAS-ESR	1.3 (0.9, 1.9)	1.2 (0.9, 1.8)	1.4 (1.0, 1.9)	0.0 (− 0.2, 0.2)	0.839	0.1 (− 0.2, 0.3)	0.032	0.1 (− 0.1, 0.4)	0.022
ASDAS-CRP	1.2 (0.8, 1.6)	1.2 (0.7, 1.8)	1.3 (0.8, 1.8)	0.1 (− 0.2, 0.3)	0.051	0.0 (− 0.2, 0.3)	0.734	0.1 (− 0.2, 0.4)	0.016
BASDAI	1.4 (0.6, 2.6)	1.6 (0.6, 2.8)	1.5 (0.6, 2.6)	0.0 (− 0.3, 0.4)	0.163	0.0 (− 0.6, 0.2)	0.024	0.0 (− 0.4, 0.4)	0.958
Back pain	1.0 (0.0, 3.0)	1.0 (1.0, 3.0)	1.0 (0.0, 3.0)	0.0 (− 1.0, 1.0)	0.375	0.0 (− 1.0, 0.0)	0.072	0.0 (0.0, 0.0)	0.324
Peripheral pain/swelling	1.0 (0.0, 2.0)	1.0 (0.0, 2.0)	1.0 (0.0, 3.0)	0.0 (0.0, 1.0)	0.362	0.0 (− 1.0, 1.0)	0.712	0.0 (0.0, 1.0)	0.168
Duration of morning stiffness	1.0 (0.0, 2.0)	1.0 (0.0, 2.0)	1.0 (0.0, 2.0)	0.0 (0.0, 0.0)	0.830	0.0 (0.0, 0.0)	0.676	0.0 (0.0, 0.0)	0.469
Patient global assessment	1.0 (0.0, 3.0)	1.0 (0.0, 2.0)	2.0 (0.0, 3.0)	0.0 (− 1.0, 1.0)	0.767	0.0 (0.0, 1.0)	0.561	0.0 (0.0, 1.0)	0.394
Fatigue	2.0 (1.0, 4.0)	2.0 (1.0, 4.0)	2.0 (1.0, 3.0)	0.0 (− 1.0, 1.0)	0.369	0.0 (− 1.0, 0.0)	0.016	0.0 (− 1.0, 1.0)	0.204
Areas of localized tenderness	1.0 (0.0, 2.0)	1.0 (0.0, 3.0)	1.0 (0.0, 3.0)	0.0 (− 1.0, 1.0)	0.336	0.0 (− 1.0, 1.0)	0.202	0.0 (− 1.0, 1.0)	0.713
Degree of morning stiffness	1.0 (0.0, 3.0)	1.0 (0.0, 2.0)	1.0 (0.0, 3.0)	0.0 (0.0, 0.0)	0.189	0.0 (0.0, 0.0)	0.559	0.0 (0.0, 1.0)	0.680

All patients with AS were assessed using the EMRMS between Feb 7 and Oct 16, 2021.

Pre-COVID-19-wave phase: 6th–17th week of 2021 (February 7–May 1, 2021).

COVID-19-wave phase: 18th–29th week of 2021 (May 2–July 24, 2021).

Post-COVID-19-wave phase: 30th–41st week of 2021 (July 25–October 16, 2021).

*The weekly calculations are based on WHO’s year-week comparison table, where each week includes all 7 days and the first week of each year does not necessarily include January 1.

^#^Kolmogorov–Smirnov p value < 0.05, Wilcoxon Signed Rank Test is used.

### Clinical remission in AS patients who assessed all three phases

Compared before and after COVID-19 wave phases, the number of AS patients with ASDAS-ESR and ASDAS-CRP < 1.3 were decreased but without significantly (60 vs. 52, p = 0.088; 68 vs. 63, p = 0.368) (Table 4).

**Table 4:** The number of AS patients in different disease activity assessed in all three phases (n = 126).

	Pre-COVID-19-wave phase (A)	COVID-19-wave phase (B)	Post-COVID-19-wave phase (C)	(B)-(A) McNemar p value	(C)-(B) McNemar p value	(C)-(A) McNemar p value
**ASDAS-ESR**						
ASDAS ESR < 1.3	60 (47.6)	64 (50.8)	52 (41.3)	0.394	0.019	0.088
1.3 ≤ ASDAS ESR < 2.1	47 (37.3)	36 (28.6)	50 (39.7)	0.048	0.031	0.612
2.1 ≤ ASDAS ESR ≤ 3.5	17 (13.5)	24 (19.0)	22 (17.5)	0.071	0.637	0.197
3.5 < ASDAS ESR	2 (1.6)	2 (1.6)	2 (1.6)	1.000	1.000	1.000
**ASDAS-CRP**						
ASDAS CRP < 1.3	68 (54.0)	64 (50.8)	63 (50.0)	0.480	0.819	0.369
1.3 ≤ ASDAS CRP < 2.1	39 (31.0)	40 (31.7)	41 (32.5)	0.879	0.862	0.752
2.1 ≤ ASDAS CRP ≤ 3.5	18 (14.3)	19 (15.1)	19 (15.1)	0.827	1.000	0.808
3.5 < ASDAS CRP	1 (0.8)	3 (2.4)	3 (2.4)	0.157	1.000	0.317
**BASDAI**						
0 ≤ BASDAI < 3	101 (80.2)	96 (76.2)	99 (78.6)	0.225	0.439	0.480
3 ≤ BASDAI < 4	13 (10.3)	11 (8.7)	11 (8.7)	0.617	1.000	0.564
4 ≤ BASDAI < 6	6 (4.8)	13 (10.3)	10 (7.9)	0.020	0.405	0.206
6 ≤ BASDAI	6 (4.8)	6 (4.8)	6 (4.8)	1.000	1.000	1.000

All patients with AS were assessed using the EMRMS between Feb 7 and Oct 16, 2021.

Pre-COVID-19-wave phase: 6th–17th week of 2021 (February 7–May 1, 2021).

COVID-19-wave phase: 18th–29th week of 2021 (May 2–July 24, 2021).

Post-COVID-19-wave phase: 30th–41st week of 2021 (July 25–October 16, 2021).

*The weekly calculations are based on WHO’s year-week comparison table, where each week includes all 7 days and the first week of each year does not necessarily include January 1.

Compared with post COVID-19 wave phases, both numbers of ASDAS-ESR and ASDAS-CRP score increased from < 1.3 to ≥ 1.3 more than decreased from ≥ 1.3 to < 1.3 (15 vs. 7; 18 vs. 13) in pre COVID-19 period; (19 vs. 7; 10 vs. 9) during COVID-19 period (Table 5).

**Table 5:** The alteration of AS patients numbers in different disease activity scores assessed in all three phases (n = 126).

		COVID-19-wave phase (B)		Post-COVID-19-wave phase (C)	
		ASDAS-ESR < 1.3	ASDAS-ESR ≥ 1.3	ASDAS-ESR < 1.3	ASDAS-ESR ≥ 1.3
Pre-COVID-19-wave phase (A)	ASDAS-ESR < 1.3	51 (85.0)	9 (15.0)	45 (75.0)	15 (25.0)
	ASDAS-ESR ≥ 1.3	13 (19.7)	53 (80.3)	7 (10.6)	59 (89.4)
COVID-19-wave phase (B)	ASDAS-ESR < 1.3			45 (70.3)	19 (29.7)
	ASDAS-ESR ≥ 1.3			7 (11.3)	55 (88.7)

		COVID-19-wave phase (B)		Post-COVID-19-wave phase (C)	
		ASDAS-CRP < 1.3	ASDAS-CRP > = 1.3	ASDAS-CRP < 1.3	ASDAS-CRP > = 1.3
Pre-COVID-19-wave phase (A)	ASDAS-CRP < 1.3	50 (73.5)	18 (26.5)	50 (73.5)	18 (26.5)
	ASDAS-CRP ≥ 1.3	14 (21.4)	44 (75.9)	13 (22.4)	45 (77.6)
COVID-19-wave phase (B)	ASDAS-CRP < 1.3			54 (84.4)	10 (15.6)
	ASDAS-CRP ≥ 1.3			9 (14.5)	53 (85.5)

All patients with AS were assessed using the EMRMS between Feb 7 and Oct 16, 2021.

Pre-COVID-19-wave phase: 6th–17th week of 2021 (February 7–May 1, 2021).

COVID-19-wave phase: 18th–29th week of 2021 (May 2–July 24, 2021).

Post-COVID-19-wave phase: 30th–41st week of 2021 (July 25–October 16, 2021).

* The weekly calculations are based on WHO’s year-week comparison table, where each week includes all 7 days and the first week of each year does not necessarily include January 1.

## Discussion

The present study revealed the influence of COVID-19 pandemic on health behavior and disease activity in patients with AS through EMRMS. Our results indicate that the disease activity of AS worse after an increase in the number of COVID-19 cases in Taiwan. A cohort of AS patients were assessed for disease activity before, during, and after the COVID-19 wave via EMRMS, the obtained ESR, ASDAS-ESR, CRP, ASDAS-CRP, and BASDAI data indicated variations in disease activity in the patients with AS, which was consistent with the results in prior literature^8,10–12^, ESR, ASDAS-ESR, CRP, and ASDAS-CRP increased in post COVID-19 wave, and our study also found AS patients with clinical remission (ASDAS-CRP < 1.3) decreased in post COVID-19 wave. This highlight of this study is to apply EMRMS application on intelligent device to assess PROs with clinical laboratory data combination and disease activity of AS patient for COVID-19 related social isolation and distancing^20^.

Rheumatologists at TCVGH started routinely assessing the ASDAS, BASDAI, medications used, and clinical outcomes of all patients with AS using the EMRMS in November 2016. During the COVID-19 wave, most communication was carried out online through social media, websites, or apps. COVID-19 has been associated with high levels of psychological distress, especially at the early stage of the pandemic, because of the lack of information on COVID-19, challenges in communication with healthcare professionals and access to medical care, and ineffectiveness of media in raising public awareness^21–24^. Individuals with axSpA exhibited high levels of stress and anxiety and considerably high disease activity levels, although they were below clinically significant levels. ASDAS-ESR and ASDAS-CRP combined PROs and clinical laboratory data were both highly correlated with disease activity. CRP increased earlier than ESR did and then rapidly decreased^25^. BASDAI entirely depends on PROs which reporting by patients (or the patient’s caregiver) and is not an objective measure of inflammation; therefore, BASDAI may be unable to capture disease status as effectively as clinical assessments, and PROs cannot replace clinical examination totally. This can be explained that ESR, ASDAS-ESR, CRP, and ASDAS-CRP increased in post COVID-19 wave, whereas BASDAI slightly decreased during the COVID-19 wave, the major factor causing this result probably comes from the change of laboratory inflammatory index.

A notable limitation of this study is that we could only evaluate patients with AS whose disease activities were regularly assessed using the EMRMS through smartphone applications during the COVID-19 pandemic. The results obtained for the subgroup that did not use smartphone apps might be underestimated. Another limitation of the study is that it is restricted to Taiwanese patients only, and the results may not be applicable to other countries or to individuals who are not of East Asian descent.

## Conclusions

This single center study revealed the health behavior and disease activity of patients with AS changed during the COVID-19 pandemic, as indicated by an analysis of both the laboratory-assessed inflammatory index and PROs using the EMRMS application at TCVGH. Our findings suggest that AS disease activity worse probably comes from laboratory data majorly after COVID-19 wave. Further clinical studies are needed to confirm our findings and elucidate the underlying etiology.

## Data Availability

All data generated or analysed during this study are included in this published article.
